# Graphene mesosponge: a novel material for the sequestration of azo dyes in water

**DOI:** 10.1016/j.jare.2025.11.027

**Published:** 2025-11-14

**Authors:** Amrita Jain, Chandini Kumar, Peter Škorňa, Hirotaka Nakatsuji, Hirotomo Nishihara, Tamas Szabo, Olena Ivashchenko, Monika Michalska, Eva Scholtzova

**Affiliations:** a Institute of Fundamental Technological Research, Polish Academy of Sciences, Pawińskiego 5B, 02-106 Warsaw, Poland; b Institute of Inorganic Chemistry, Slovak Academy of Sciences, Dúbravská cesta 9, 845 36 Bratislava, Slovakia; c Institute of Multidisciplinary Research for Advanced Materials, Tohoku University, 2-1-1 Katahira, Aoba-ku, Sendai 980-8577, Japan; d Advanced Institute for Materials Research (AIMR-WPI), Tohoku University, 2-1-1 Katahira, Aoba-ku, Sendai 980-8577, Japan; e Department of Physical Chemistry and Materials Science, University of Szeged, Rerrich Béla Tér. 1, H-6720 Szeged, Hungary; f NanoBioMedical Centre, Adam Mickiewicz University, 61-614, Poznań, Poland; g Department of Chemistry and Physico-Chemical Processes, Faculty of Materials Science and Technology, VSB-Technical University of Ostrava, 17. listopadu 2172/15, 708 00 Ostrava-Poruba, Czech Republic

**Keywords:** Methylene Blue, Congo Red, Azo dyes, Adsorption, Carbon, AIMD modelling

## Abstract

Azo dyes are synthetic organic compounds widely used in industries such as textiles, printing, and pharmaceuticals. Due to their chemical stability and extensive usage, they are significant environmental pollutants, especially in wastewater. This study investigates the removal of azo dyes from aqueous solutions using a graphene mesoporous sponge (GMS), which is a high-surface sponge-like mesoporous structure predominantly composed of single-layer graphene walls with active sites for dye adsorption. Methylene Blue (MB) and Congo Red (CR) were used to evaluate adsorption performance in aqueous solutions simulating industrial effluents. The adsorption behavior, capacity, and kinetics were studied experimentally, showing that GMS efficiently removes both dyes. Complete decolorization occurred in 30–35 min for both dyes at an initial concentration of 1 mg L^−1^. A relatively small quantity of GMS successfully removed significant amounts of dye, highlighting its potential to minimize material usage. This efficiency enhances the process’s economic feasibility while contributing to environmental sustainability by reducing the carbon footprint of adsorbent synthesis and usage. These results indicate that GMS is a promising material for wastewater treatment. The experimental results are also aligned with ab-initio molecular dynamics (AIMD) calculations, and they confirm the superior performance of GMS over pristine graphene in dye adsorption.

## Highlights

• GMS adsorbent attained 100 % removal of azo dye in a mere 30–35 min.
• Mesoporous sponge-like GMS provides a large surface area for the adsorption of dye.
• Low quantities of GMS eliminated huge volumes of dye, minimizing the use of material.
• GMS demonstrated better adsorption of dye than pure graphene through AIMD.
• Effective removal of dye enhances sustainability and decreases carbon footprint.

## Graphical abstract

**Figure:**
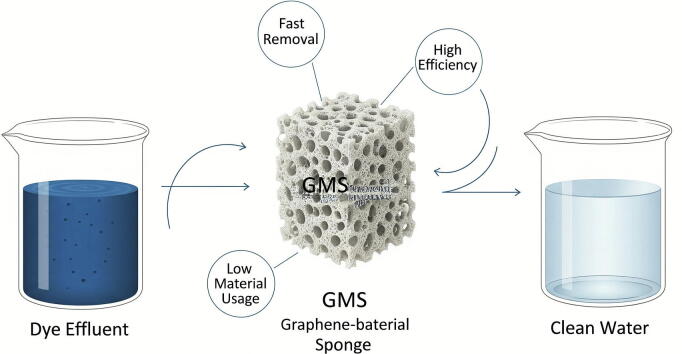

## Introduction

The excessive use of azo dyes in various industries like textile, leather, paper, printing, and cosmetics results in significant environmental pollution due to frequent discharge into wastewater. Their complex molecular structures make dyes highly stable and resistant to degradation in water, which poses challenges to their removal [1]. Azo dyes and their by-products formed after degradation harm both flora and fauna, and exhibit mutagenic or carcinogenic effects on the human body [2]. Even small concentrations of these dyes in water are problematic because they impart a visible coloration, which hinders critical ecological processes such as sunlight penetration, suppressing photosynthesis, and impairing the growth of aquatic organisms, making water unappealing and interfering with its natural properties, also disrupting the solubility of gases in water [[3], [4], [5]]. So far, almost 100,000 dyes have been commonly used and commercially available in the market with an approximate annual production of more than 7x10^5^ tons/year [6,7]. However, the worldwide total dye consumption is around 100,000 tons/year, out of which almost 100 tons/year of dyes are entering the environment through the wastewater discharge [[8], [9], [10]]. Methylene Blue (MB) and Congo Red (CR) are common examples of industrial effluent dyes. MB, a cationic dye, is not considered highly toxic but can have adverse effects on humans and animals. Exposure may cause eye irritation, and ingestion can irritate the gastrointestinal tract, leading to symptoms such as nausea, vomiting, and diarrhea [11]. On the contrary, CR, an anionic dye, contains benzidine, a compound classified as a carcinogen, which has various toxic effects on organisms at different trophic levels [12]. While the use of CR in the textile industry has largely been discontinued [13], it may still be a local contaminant in certain countries that do not recognize it as a hazardous material. Given their detrimental impacts on the environment and human health, the removal of azo dyes from wastewater is of paramount importance for ensuring ecological safety and public health.

Conventional wastewater treatment methods typically involve a combination of physical, chemical, and biological processes, categorized into preliminary, primary, secondary, and tertiary levels of treatment. Despite the effectiveness of conventional methods, they often struggle with the complete degradation or removal of persistent pollutants, such as azo dyes, pharmaceutical drugs, and pesticides. In recent years, significant attention has been directed toward advanced oxidation processes (AOPs) as promising alternatives for treating refractory pollutants. These methods include the Fenton reaction [14,15], photolysis [[15], [16], [17]], photo-Fenton [15,16,[18], [19], [20]], photocatalysis [17,[21], [22], [23], [24], [25], [26], [27], [28]], ozonation [22,29], sonochemistry [15,30], microwave-assisted oxidation [31,32]. Fenton-like reactions (involving particle systems) [33,34], and adsorption [[35], [36], [37], [38], [39], [40]].

While AOPs offer various advantages, with their own set of limitations. For instance, sonochemical processes often require high energy input, making them energy-intensive; strict pH requirements constrain Fenton reactions [15]; photo-Fenton processes involve high water consumption; and highly effective adsorption methods can be cost-prohibitive for large-scale implementation. Despite these challenges, the ongoing development and optimization of AOPs continue to enhance their feasibility and effectiveness for wastewater treatment.

Various adsorbents for dye removal in wastewater treatment include low-cost options like agricultural waste (orange peels, leaves) and natural clays (bentonite, zeolites) [[41], [42], [43], [44]]. Polymer-based adsorbents such as chitosan-based nanocomposite gels, graphene oxide hydrogels, and cellulose-based materials offer high adsorption capacities and tunable properties [[45], [46], [47]]. Nanomaterials, including carbon nanotubes, graphene, and metal–organic frameworks (MOFs), provide high surface areas and unique adsorption capabilities [48,49]. Geopolymer adsorbents, synthesized from industrial waste, offer a cost-effective solution for heavy metal removal [50]. Magnetic adsorbents facilitate easy separation, while composite adsorbents combine materials for enhanced performance [51,52]. The selection depends on dye type, wastewater composition, cost, and environmental factors, with ongoing research focusing on eco-friendly, high-capacity adsorbents [[53], [54], [55]]. Elevated-temperature adsorption, utilizing materials like MOFs and zeolites, is also employed in specific applications [56]. This variety of adsorbents addresses the critical need for efficient and sustainable wastewater treatment [57]. Specially, in dye adsorption, particularly for methylene blue (MB, cationic) and Congo red (CR, anionic), both mechanisms present challenges. Physisorption, relying on weak van der Waals forces, often results in low capacity and poor selectivity, making the process inefficient and reversible. Chemisorption, while stronger and more specific, creates an irreversible bond. This makes adsorbent regeneration nearly impossible, increasing costs and creating secondary waste, while also sometimes being too slow for practical wastewater treatment.

Carbon materials, such as activated carbon, carbon nanotubes, graphene, and biochar, are frequently employed due to their adsorption capacity in wastewater treatment. This efficacy stems from their substantial surface area, porosity, and ability to attract and retain dye molecules. Activated carbon [58,59], with its extensive pore network, is especially effective in removing organic pollutants, including dyes, from water. Carbon nanotubes [60], with their distinctive structure and large surface area, exhibit exceptional adsorption capacities, rendering them highly efficient for dye removal. Graphene-based materials [61] are highly recommended due to their substantial surface area and their capacity for strong interactions with dye molecules. Biochar, a by-product of organic waste, is a sustainable and cost-effective alternative, offering a porous structure that enhances dye adsorption while being environmentally friendly [[62], [63], [64]]. The employment of these carbon materials has been shown to yield substantial benefits, including high efficiency, recyclability, and the capacity for modification to target specific types of dyes. This versatility renders them highly valuable in water treatment.

In this study, we propose the application of GMS in wastewater treatment for dye removal through the adsorption process. The synthesis and characterization of GMS were published in our previous article [65]. GMS is a synthesized material characterized as a sponge-like mesoporous framework with a mean pore size of 5.8 nm, consisting predominantly of coalesced single-layer graphene sheets. This unique framework structure effectively prevents graphene sheet stacking, resulting in an exceptionally large specific surface area of 1940 m^2^ g^−1^. Additionally, the continuous graphene framework of GMS contains a minimal number of edge sites, significantly enhancing its oxidation resistance. These characteristics have not been simultaneously achieved in any previously reported nanoporous carbon materials.

Furthermore, the seamless single-layer graphene-based sponge-like structure endows the GMS with remarkable elasticity and mechanical toughness. These mechanical properties are unprecedented among mesoporous materials with small pore sizes (<10 nm), as conventional materials typically rely on rigid, brittle frameworks. The exceptional elasticity of GMS enables it to behave as a nanoscale sponge capable of reversible deformation [66]. This property facilitates in situ adsorption control by applying mechanical force, which allows for dynamic and efficient adsorption processes. The unique combination of GMS properties, derived from its continuous single-layer graphene walls devoid of edge sites, makes it a highly innovative material for environmental applications.

Although there are various methods for the removal of dyes from aqueous solutions or wastewater, most are based on large amounts of adsorbents, which complicates processes and increases material consumption [[67], [68], [69], [70]]. The proposed approach using GMS overcomes these limitations by enabling the removal of high concentrations of dye from aqueous media with only a minimal amount of adsorbent, significantly reducing material requirements and operational costs. This not only enhances process efficiency but also contributes to environmental sustainability by reducing the carbon footprint associated with the treatment process.

## Experimental details

### Materials and methods

**Fig. 1:**
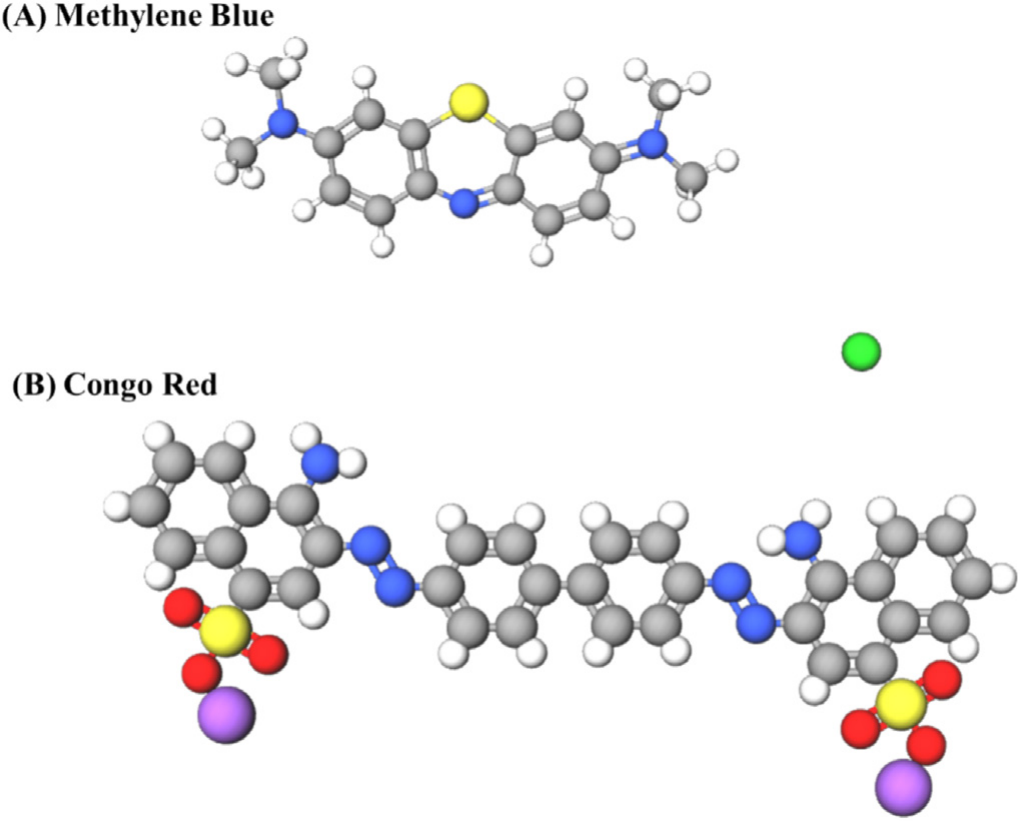
Chemical structure of (A) Methylene Blue (C_16_H_18_CIN_3_S) and (B) Congo Red (C_32_H_22_N_6_Na_2_O_6_S_2_) dyes.

Both azoic dyes, Methylene Blue and Congo Red, with molecular weights of 319.85 g mol^−1^ and 696.66 g mol^−1^, respectively, were procured from Sigma-Aldrich, Poland, and used as received. The chemical structures of both dyes are shown in Fig. 1 [71,72]. The maximum adsorption wavelengths for MB and CR, as determined using a spectrophotometer, are 663 nm (∼665 nm) and 499 nm (∼500 nm), respectively [33,73]. To prepare the solutions, a stock solution of 1000 mg L^−1^ for each dye was prepared separately in amber bottles and stored at 15 °C in a refrigerator to prevent evaporation and maintain consistent concentrations. For experimental studies, these stock solutions were subsequently diluted with deionized (DI) water to achieve concentrations ranging from 10 to 200 mg L^−1^ [34]. A concentration of 10 mg L^−1^ was used for batch studies, while a concentration of 100 mg L^−1^ was selected for kinetics studies.

### Synthesis of GMS

The GMS was synthesized using a template-assisted chemical vapor deposition (CVD) technique. Aluminum oxide (Al_2_O_3_, SBa-200) nanoparticles were employed as nanosized spherical substrates for carbon deposition. Methane (CH_4_) was used as the carbon source, and the deposition was conducted at 900 °C for 2 h. During this process, the surface of the nanoparticles turned completely black, indicating a successful carbon deposition. Carbon deposition occurred exclusively on the surface of the Al_2_O_3_ nanoparticles due to their catalytic activity, resulting in a uniform thin carbon layer that covers the entire surface of the nanoparticles.

Subsequently, Al_2_O_3_ nanoparticles were removed through chemical etching by hydrofluoric acid, leaving behind a carbon mesosponge with a spherical mesoporous structure. The carbon structure was further subjected to high-temperature heat treatment at 1800 °C for 1 h in Ar (1 atm), which facilitated the crystallization of the carbon sheets into graphene. The resultant material, which is similar to a nanosponge, was termed Graphene Mesosponge (GMS).

Please note: For detailed synthesis procedures, refer to the article published by our group elsewhere [74].

### Computational modeling and methodology

**Fig. 2:**
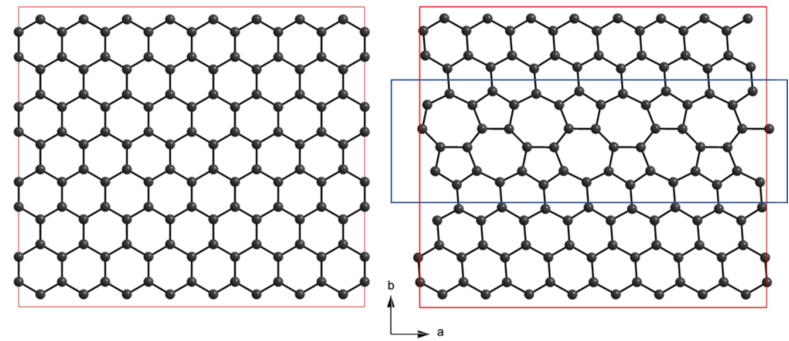
Structure of pristine graphene, G6 (left), and graphene with topological defects, G57 (right).

The additional information regarding the way of interaction of the studied azo dyes with the surface of the adsorbent material and the stability of azo dye–GMS material hybrid structures was evaluated at the atomic level using a modeling approach. For the study, the idealized model of GMS material (G57) was prepared from the ideal graphene structure containing 6-membered rings – G6 (Fig. 2), involving topological defects with the 5, 7-membered rings in the structure detected in the GMS structure [74] to have a compromise between the real structure and computational demand. The G6 model was used to compare adsorption effectiveness with the G57 model (GMS). Hybrid structures with azo dyes were labelled MB-G6, CR-G6, MB-G57 and CR-G57.

The initial computational cell of the proposed models had lattice vectors *a* = 19.648 Å and *b* = 34.032 Å. The *c* vector had an initial size of 30 Å for all four models to preserve the vacuum sorption surface models.

The computational method based on the Density functional theory (DFT) implemented in the *ab initio* VASP program [75] was used for optimization involving the D3 scheme for correction of dispersion forces [76] at the *gamma* point because of the model's size, using an energy cut-off of 400 eV. Further, the *ab initio* molecular dynamics (AIMD) was applied. The Verlet velocity algorithm [77] was chosen for a numerical solution of equations of motion using a time step of one fs. The finite temperature calculations were carried out on a canonical (NVT) ensemble, applying the Nosé-Hoover thermostat procedure [78,79] at the simulation temperature of 300 K to equilibrate the structure and use the MD length of ten ps.

The adsorption energy, *E*_ads_, as a measure of azo dyes-G6/G57 hybrid systems’ stability, was calculated from the respective total energies, *E*_tot_, according to the scheme*E*_ads_ = Σ*E*_tot(products)_ − Σ *E*_tot(reactants)_, where the lower the *E*_ads_ value, the higher the hybrid system’s stability.

## Results and discussion

Initially, stock solutions of known concentrations (1000 mg L^−1^) were prepared for both dyes. Further, these stock solutions were diluted to obtain 1–200 mg L^−1^ concentrations in deionized (DI) water. The diluted solutions were analyzed using a UV–visible spectrophotometer to generate calibration curves by plotting concentration against absorbance at 665 nm for Methylene Blue (MB) and 570 nm for Congo Red (CR), respectively.

### Characterization and instrument details

The morphology of the GMS before and after adsorption was characterized using an EM/FIB-Zeiss Crossbeam 350 (Germany). FTIR transmittance spectra were obtained using an FT/IR-4700 spectrometer equipped with a TGS detector (JASCO). Samples were prepared using potassium bromide as a matrix material and were mixed at a proportion of approximately 1 mg of the sample to 200 mg KBr. Pellets were prepared using Specac® Atlas 15 T manual hydraulic press under a pressure of 10 tons/cm^2^ with a barrel 13 mm in diameter. Measurements were performed at room temperature. For each spectrum, 64 scans in the spectral range of 4000–400 cm^−1^ were taken with a resolution of 4 cm^−1^. Data was processed using the Jasco software package; baseline correction was applied. X-ray diffraction (XRD) analysis was performed by a Philips PW1830 diffractometer operating with an X-ray tube emitting CuKα radiation, with 40 kV acceleration voltage at 30 mA current to evaluate changes in the material's crystallinity before and after dye adsorption. Size of the GMS was determined in a Nano ZS dynamic light scattering (DLS) apparatus (Malvern Instruments, Malvern, Worcestershire, UK) with a 4 mW He-Ne laser source (λ = 633 nm). Additionally, an Edinburgh FS5 spectrophotometer was employed to obtain the absorption spectra of the azo dyes at their maximum wavelength.

### Batch adsorption study

The batch adsorption study was conducted using variable adsorbent concentrations. The experiments were performed in a stirred conical flask, where the adsorbent concentration was varied from 0.1 to 1 % (w/v) with a dye concentration of 20 mg L^−1^ for both dyes. The concentration of 20 mg L^−1^ was selected because it falls within the typical range of azo dye concentrations (5–30 mg L^−1^) found in industrial effluents. The reaction solution was mechanically stirred at room temperature (20 °C), and samples were collected at different time intervals using a micropipette. Immediately after sampling, the samples were centrifuged at 5000 RPM for 10 min to prevent further adsorption during contact time. The supernatant was analyzed for dye concentration using UV–visible absorption spectroscopy with an FS5 spectrophotometer at 665 nm for Methylene Blue (MB) and 500 nm for Congo Red (CR).

**Fig. 3:**
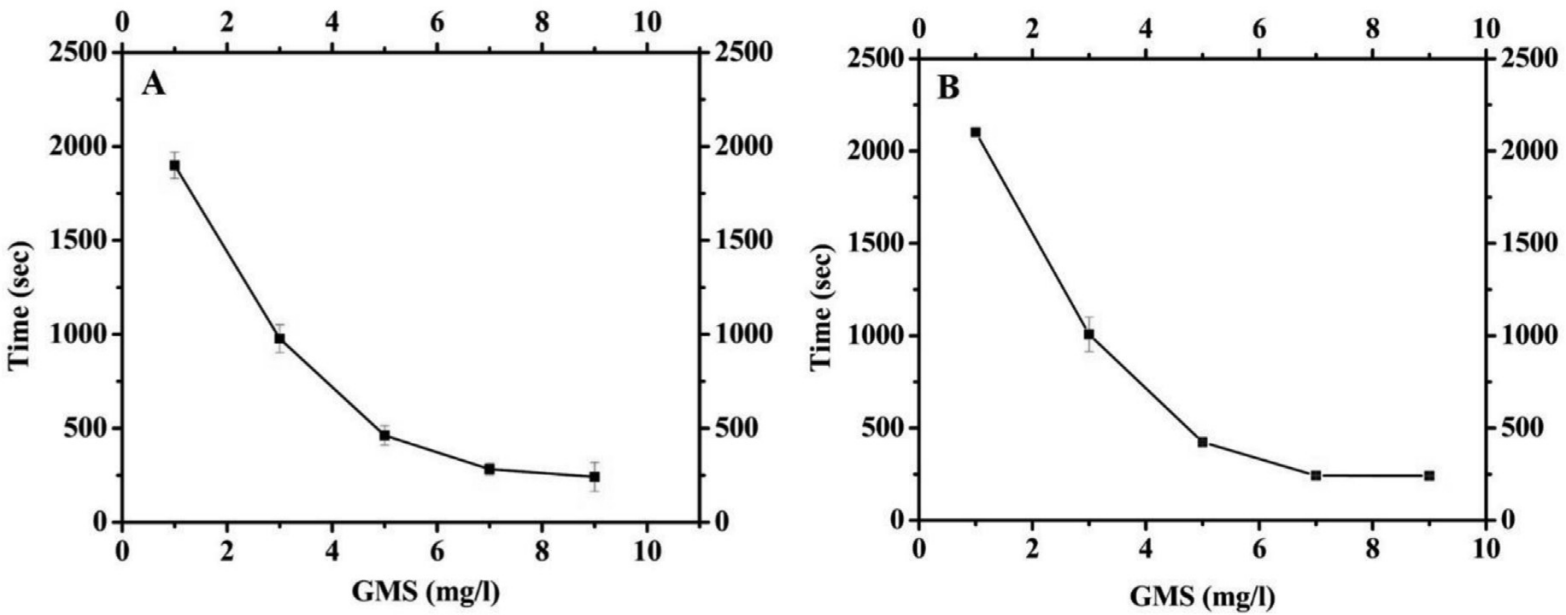
Effect of adsorbent concentration on the dye removal; A) For MB dye and B) For CR.

Note: All experiments were repeated three times to ensure reliability and accuracy. The average values from the triplicate measurements were used for data analysis, with deviations from the average concentration falling within the range of 5–10 %.

Fig. 3 illustrates the time required for complete dye removal as a function of adsorbent concentration. GMS demonstrates rapid adsorption of Methylene Blue (MB) (Fig. 3A), even at low adsorbent concentrations. However, the adsorption of Congo Red (CR) (Fig. 3B) took slightly longer compared to MB. This difference suggests that GMS has a more favorable adsorption affinity for cationic dyes like MB, likely due to the availability of free functional groups (–OH/–CH) on the GMS surface. In contrast, the adsorption of CR, which involves interactions with (–NH/–CN) functional groups, proceeds at a slower rate of adsorption. Ghaedi et al. 2013, showed the adsorption of MB using activated carbon prepared with a low-cost locally available material, peanut stick wood, and demonstrated that the activated carbon takes almost 45 min to reach almost 95 % removal of dye [68]. Ojedokun, et al. 2017, demonstrate the use of activated carbon based on guava leaf for CR adsorption and reveal that CR adsorption takes almost 150 min to fully adsorb with the initial concentration of 10 mg L^−1^ [80]. However, the synthesized GMS takes less time compared to activated carbon. Also, the amount of 10 mg L^−1^ of activated carbon and 1 mg L^−1^ of the GMS, respectively, makes it more effective with less carbon footprint in the environment.

### Adsorption kinetic studies

The adsorption kinetic studies were carried out, and the adsorbent–adsorbate solution was collected at specific intervals, centrifuged to avoid direct contact time during the measurement, and the concentration of the solution was measured using a UV-spectrophotometer. The amount adsorbed at time t, q(t) (mg/g), was calculated using Eq. (1). The adsorption kinetics of dye on adsorbent were investigated using pseudo-first-order and pseudo-second-order methods, respectively, to understand the real adsorption phenomena of the dye concerning GMS.

(1) $$q\left(t\right)=\frac{C_{o}-C_{e}}{W}V$$

Here, $C_{o}$ (mg L^−1^) is the initial concentration of dyes, and $C_{e}$ is the concentration of dyes once the equilibrium is reached. V (L) is the volume of MB solution in the flask, and W (g) is the mass of activated carbon used in each experiment.

The dynamics of the dye adsorption process can be described using various kinetic models. Specifically, the pseudo-first-order adsorption kinetics is calculated and validated using the linear form of Eq. (2). In contrast, the pseudo-second-order kinetics is evaluated using the linear form of Eq. (3). These models help to understand and quantify the rate and mechanism of the adsorption process.

(2) $$q\left(t\right)=q_{e}\left(1-e^{-k_{1}t}\right)$$

(3) $$q\left(t\right)=\frac{k_{2}q_{e}^{2}t}{1+k_{2}q_{e}t}$$

where $k_{1}$ (1/min) and $k_{2}$ (g/mg/min) are the pseudo-first-order and pseudo-second-order adsorption rate constants.

**Fig. 4:**
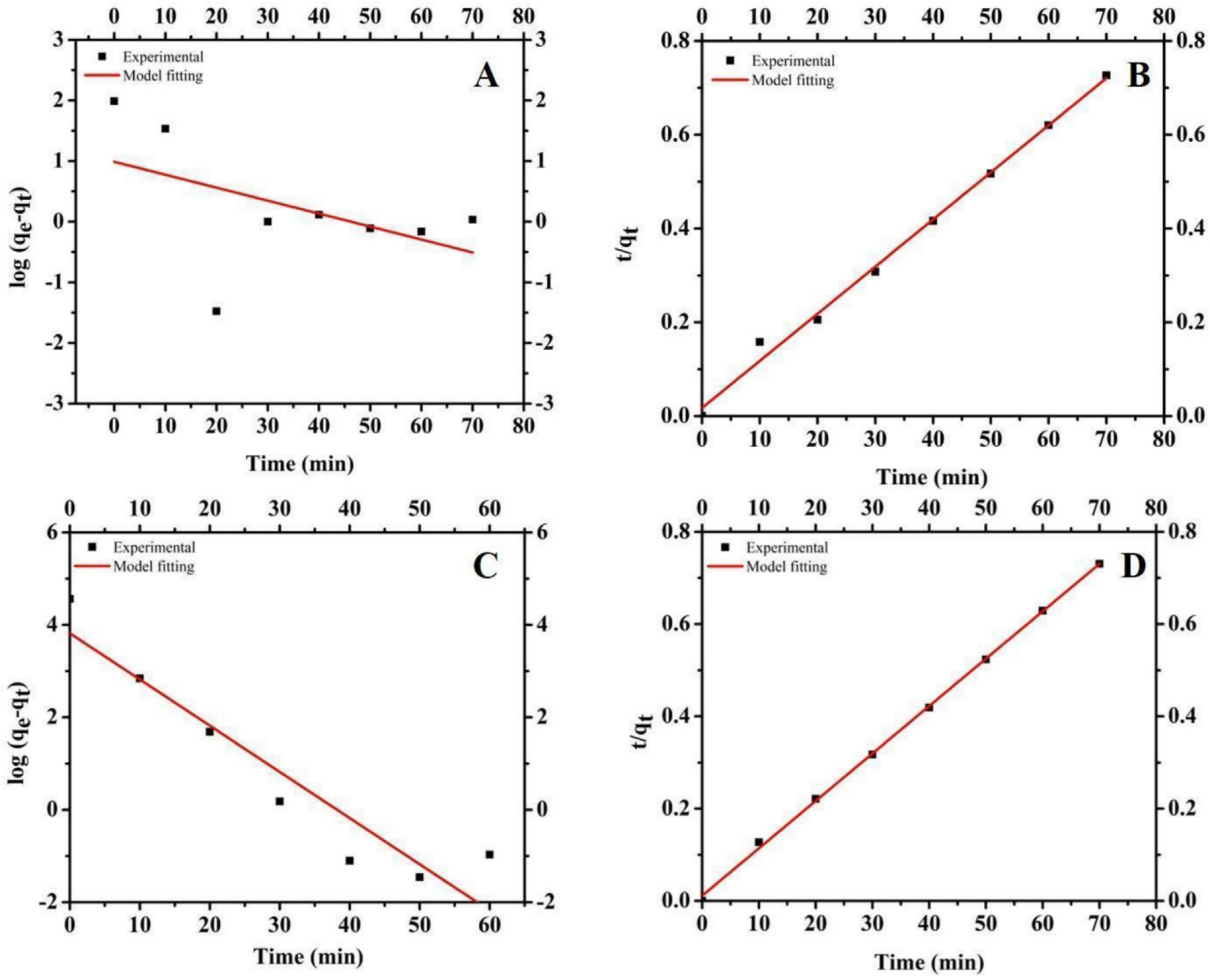
Pseudo first and second order kinetics for the adsorption; A, B for MB and C, D for CR dye, respectively.

The kinetic models were fitted to the experimental data. Fig. 4, Fig. 4 represent the kinetic model fitting for MB, and Fig. 4, Fig. 4 for CR dye, respectively, and Table 1 shows the fitting parameters of the experimental data. The results indicate that MB and CR adsorption follow pseudo-second-order kinetics, which suggests a higher amount of dye adsorption on the GMS and rapid dye uptake.

**Table 1:** Kinetic model fitting parameters for dyes.

Model	Parameter	Methylene Blue	Congo Red
Pseudo-first-order	$Q_{\mathrm{eexp}}$	97.4	95.4
	$Q_{\mathrm{ecal}}$	2.68	45.54
	$K_{1}$	0.021	0.099
	$R^{2}$	0.11	0.87

Pseudo-second-order	$Q_{\mathrm{eexp}}$	97.4	95.4
	$Q_{\mathrm{ecal}}$	99.5	97.27
	$K_{2}$	5.84 x 10^−3^	9.36 x10^−3^
	$R^{2}$	0.99	0.99

### Adsorption isotherm studies

The adsorption isotherms help to understand how the adsorption molecules are allocated between the liquid and the solid phase when the adsorption process reaches an equilibrium state. The analysis of the experimental isotherm data with the fitting of the various adsorption models is important to find a suitable model that can be useful for design procedures [81].

The applicability of different isotherm models is evaluated by comparing their correlation coefficients (R^2^ values), with higher R^2^ indicating a better fit to the experimental data.

**Fig. 5:**
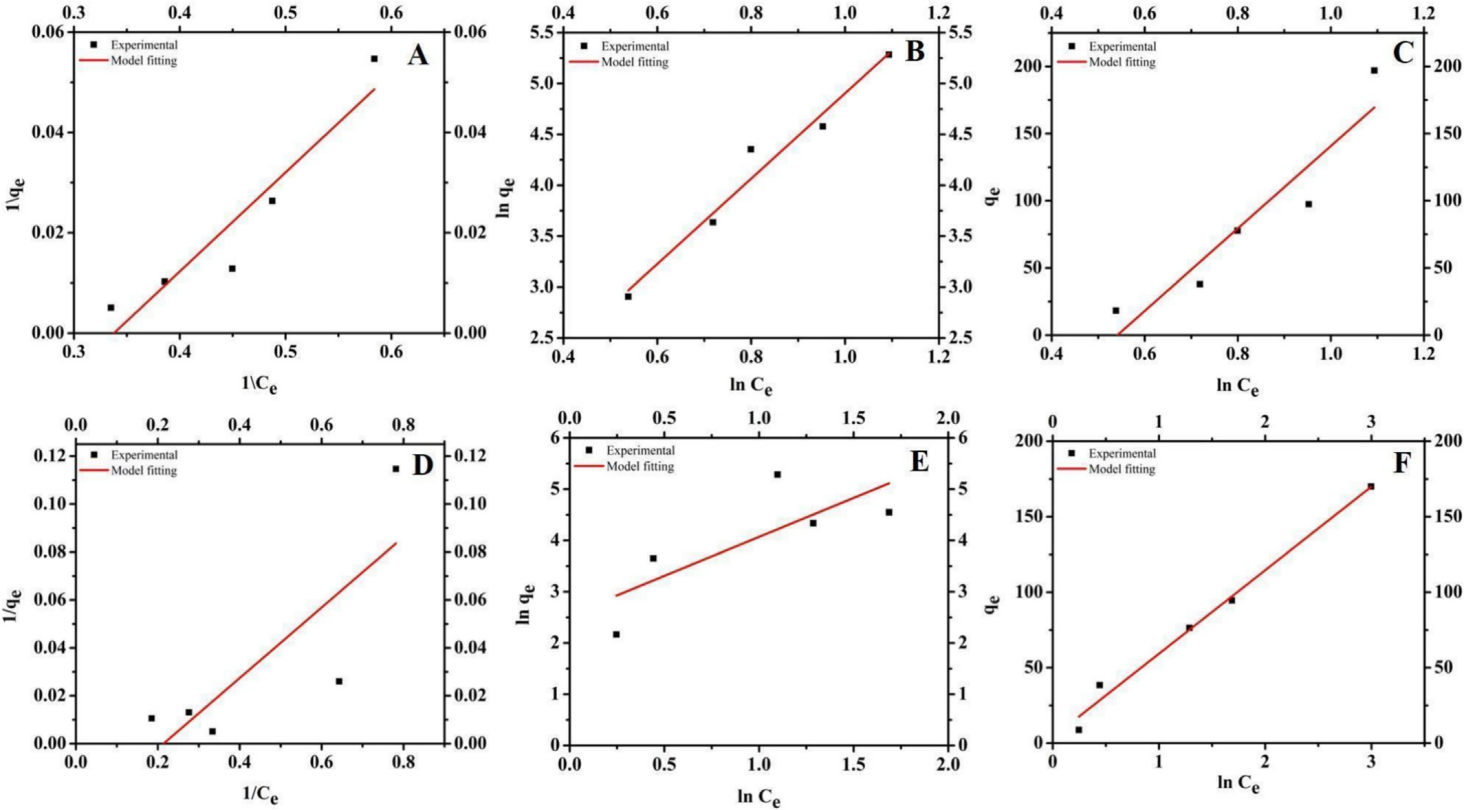
Langmuir, Freundlich, and Temkin isotherms for the adsorption of direct dye on GMS A, B, and C for MB and D, E, and F for CR, respectively.

Fig. 5 typically shows the adsorption isotherm of the MB and CR dyes on the GMS. Isotherms describe how the solute interacts with the adsorbent and help to use and optimize the adsorbent application. Adsorption isotherm studies were performed with the three well-known isotherms, Langmuir, Freundlich, and Temkin models. These models very well define the adsorption mechanism of the phases, as the correlation coefficient ranges from 0.5 to 1 for both dyes (see Table 2).

**Table 2:** Isotherm fitting model for dyes.

Model	Parameters	Methylene Blue	Congo Red
Langmuir	$Q_{m}$	14.985	31.777
	$K_{L}$	0.337	0.213
	$R^{2}$	0.86	0.56

Freundlich	$K_{f}$	5.152	354.17
	l/n	4.192	1.520
	$R^{2}$	0.95	0.46

Temkin	$b_{\mathrm{tem}}$	8.074	44.76
	$K_{T}$	1.717	1.070
	$R^{2}$	0.84	0.98

### Langmuir Isotherm

The Langmuir model was developed by Irving Langmuir in 1916 and is one of the most widely used models to describe adsorption processes, particularly in the context of gas adsorption on solid surfaces or solute adsorption from liquid solutions. It described the adsorption of a monolayer of molecules onto a homogeneous surface. Langmuir isotherm assumes monolayer adsorption onto a surface containing a finite number of adsorption sites of uniform strategies of adsorption strategies with no transmigration of adsorbate in the plane of the surface [82]. Equation (4) shows the linear mathematical form of this model.

(4) $$\frac{1}{q_{e}}=\frac{1}{Q_{m}}+\left(\frac{1}{K_{L}Q_{m}}\right)\frac{1}{C_{e}}$$

Here, where $K_{L}$ is the Langmuir constant related to the energy of adsorption (l/mg), and $Q_{m}$ is the maximum amount of adsorption corresponding to complete monolayer coverage on the surface (mg/g).

### Freundlich Isotherm

The Freundlich isotherm is a versatile and widely used model for describing adsorption on heterogeneous surfaces, especially in systems where the Langmuir assumptions do not hold. However, it is empirical and does not provide a theoretical basis for understanding adsorption mechanisms. The Freundlich isotherm model assumes heterogeneous surface energies, in which the energy term in the Langmuir equation varies as a function of surface coverage [82,83]. Equation (5) shows the linear mathematical form of this model.

(5) $$\ln q_{e}=lnK_{F}+\frac{1}{n}lnC_{e}$$

where $K_{F}$ is roughly an indicator of the adsorption capacity, and $\frac{l}{n}$ is the adsorption intensity.

### Temkin Isotherm

The Temkin adsorption isotherm is a model used to describe the adsorption of molecules onto a solid surface, considering the effects of adsorbate–adsorbate interactions and the heterogeneity of the adsorption surface. Unlike the Langmuir and the Freundlich isotherms, the Temkin model assumes that the heat of adsorption decreases linearly with increasing surface coverage due to these interactions. This makes it particularly useful for systems where adsorbate–adsorbate interactions are significant [83]. Equation (6) shows the linear mathematical form of the isotherm.

(6) $$Q_{e}=B\ln K_{T}+B\ln C_{e}$$

Here, $Q_{e}$ is the amount of dye adsorbed per unit weight of adsorbent (mg/g), $C_{e}$ the equilibrium concentration of dye solution (mg L^−1^), and B is the constant related to the free energy of adsorption (mg L^−1^).

Note: All the isotherm models used in this work are in the linear form for easy estimation.

The Freundlich isotherm model is the best fit for MB with an excellent R^2^ of 0.95. The result is strongly suggestive of the fact that the adsorbent surface is heterogeneous in nature, and the process of adsorption is non-uniform in nature. The Freundlich model is an empirical isotherm that is equivalent to a non-uniform, or heterogeneous, distribution of adsorption sites with varying affinities. This is in direct contradiction with the monolayer assumption of the Langmuir model. The fact that the Langmuir model has a comparatively lower R^2^ of 0.86 is a confirmation that the MB molecules do not adsorb onto the homogeneous surface in a pure, single layer. Instead, the adsorption energy for MB is not uniform but heterogeneous across the surface, presumably due to differences in the chemical nature or physical texture of the active sites. The Freundlich exponent value (n = 1.56) above one also indicates that the adsorption is a highly favorable process and that the dye molecules interact strongly with the adsorbent. The adsorption of CR is best described by the Temkin isotherm model with a very good R^2^ of 0.98. The outcome gives yet another perspective on the heterogeneity of the adsorbent surface. The Temkin model is different from the others in that it considers the effect of adsorbate–adsorbate interactions by assuming that the heat of adsorption decreases linearly with coverage of the surface. This means that as additional CR molecules adsorb onto the surface, they begin to interact with one another, and it becomes energetically less favorable for additional molecules to bind. This is quite a different behavior from a simple monolayer or multilayer formation described by other models. The very poor agreement of the Langmuir model (R^2^ = 0.56) for Congo Red greatly rejects the concept of a homogeneous surface and monolayer adsorption for this dye. The data also reveals a higher Temkin constant for CR compared to MB, indicating a much higher binding tendency of Congo Red for the adsorbent surface. This difference in affinity is likely to be the result of the molecular structure and charge differences of the two dyes and how they interact with the provided active sites of the adsorbent. Overall, the best-fit models for both dyes highlight that even on the same adsorbent, the process of adsorption is significantly dependent on the nature of the adsorbate. The lower Q_m_ for CR shows that GMS might achieve equilibrium much faster, making it suitable for rapid water treatment applications where short contact times are crucial. Furthermore, its optimal dose might be lower, preventing the particle aggregation often seen with higher doses of other adsorbents, thereby maintaining a higher efficiency per unit mass. This rapid, efficient performance, combined with potentially lower production costs, makes GMS a viable alternative despite its lower overall capacity.

### Interparticle diffusion analysis

The mass transfer of MB and CR into the interior of the GMS adsorbent is characterized by an intraparticle diffusion coefficient. This process, which can involve surface diffusion, pore diffusion, or a combination of both, may serve as the rate-determining step for the overall adsorption process. To describe this mass transfer mechanism, the Weber-Morris intraparticle diffusion model is applied. This model, based on Fick's second law, is widely used in adsorption studies to evaluate the rate-limiting step. The linear form of the model is expressed as:

(7) $$q_{t}=k_{\mathrm{id}}t^{0.5}+C$$

**Fig. 6:**
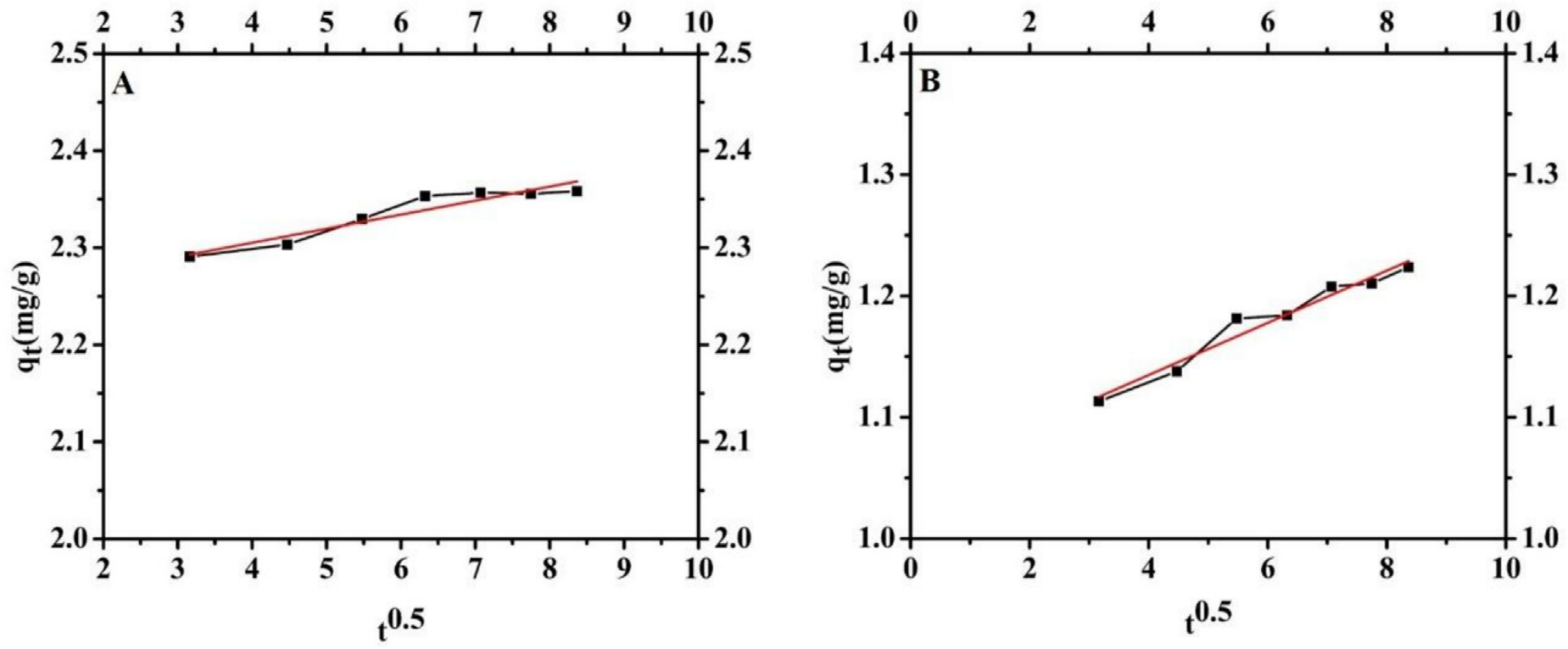
Mass transfer model analysis (A) for MB and (B) for CR.

Here, q_t_ (mg/g) is the adsorption capacity at time t (min). The intraparticle diffusion rate constant, k_id_ (mg/g·min^0.5^), and the boundary layer thickness constant, C (mg/g), are determined from the slope and intercept, respectively, of a linear plot of q_t_ versus t^0.5^ (see Fig. 6). This model suggests that intraparticle diffusion is the sole rate-controlling step if the plot of q_t_ versus t^0.5^ is linear and passes through the origin (C = 0) Ref. [19].

As illustrated in Fig. 6, the adsorption plot for MB and CR onto GMS shows linear interaction. This linear section is attributed to the intraparticle diffusion of dye molecules into the pores of the GMS adsorbent. The linearity of this section confirms that this stage of the process is controlled by diffusion.

### Adsorption mechanism

**Fig. 7:**
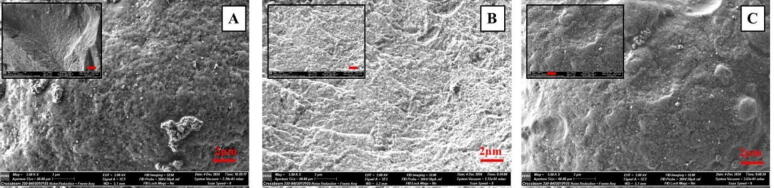
SEM images of adsorbate before and after treatment, A) pure GMS, B) after MB dye treatment, and C) after CR dye treatment.

We performed the SEM analysis to examine the morphological changes in the carbon surface before and after dye adsorption to understand the structural change in the adsorbent surface, as shown in Fig. 7. Before adsorption, the carbon surface appeared highly porous, with a rough and irregular texture, indicating the presence of well-developed micro- and mesopores. These structural features suggest a high surface area favorable for adsorption. After dye adsorption, noticeable changes were observed in the surface morphology. The previously visible pores appeared less prominent or partially blocked, suggesting the successful attachment of dye molecules onto the carbon surface and within its pores. Additionally, the surface exhibited a smoother appearance, likely due to the formation of a dye layer that covered the carbon structure. In some cases, agglomeration of dye particles was evident, particularly at higher dye concentrations, further confirming the adsorption process. These morphological changes support the efficiency of the carbon material in dye removal and align with the adsorption performance observed in other characterization techniques.

Further, to get more insight, we performed the DLS and ELS analysis of the GMS before and after the treatment. The corresponding results are shown in Table 3. The size measurements were performed using Dynamic Light Scattering (DLS) techniques. The instrument utilizes a He/Ne laser with a wavelength of 633 nm as the light source, and measurements were taken at a scattering angle of 173°. The number of parallel measurements and runs for each measurement was optimized using the integrated algorithm of the Zetasizer Nano software (v3.30) to ensure accuracy and reliability.

**Table 3:** DLS and ELS results of GMS before and after dye adsorption.

S. No.	Sample	T (°C)	Size (nm)
1	GMS	25	2016 ± 606
2	GMS + CR	25	3032 ± 433
3	GMS + MB	25	5578 ± 2900

The continuous increase in the size of the GMS indicates the formation of aggregates, which occurs due to changes in surface charge, leading to particle aggregation. This size increase is approximately 1.5 times for the anionic dye and 2.8 times for the cationic dye, clearly demonstrating the more favorable and faster adsorption of the cationic dye onto the GMS surface compared to the anionic dye.

**Fig. 8:**
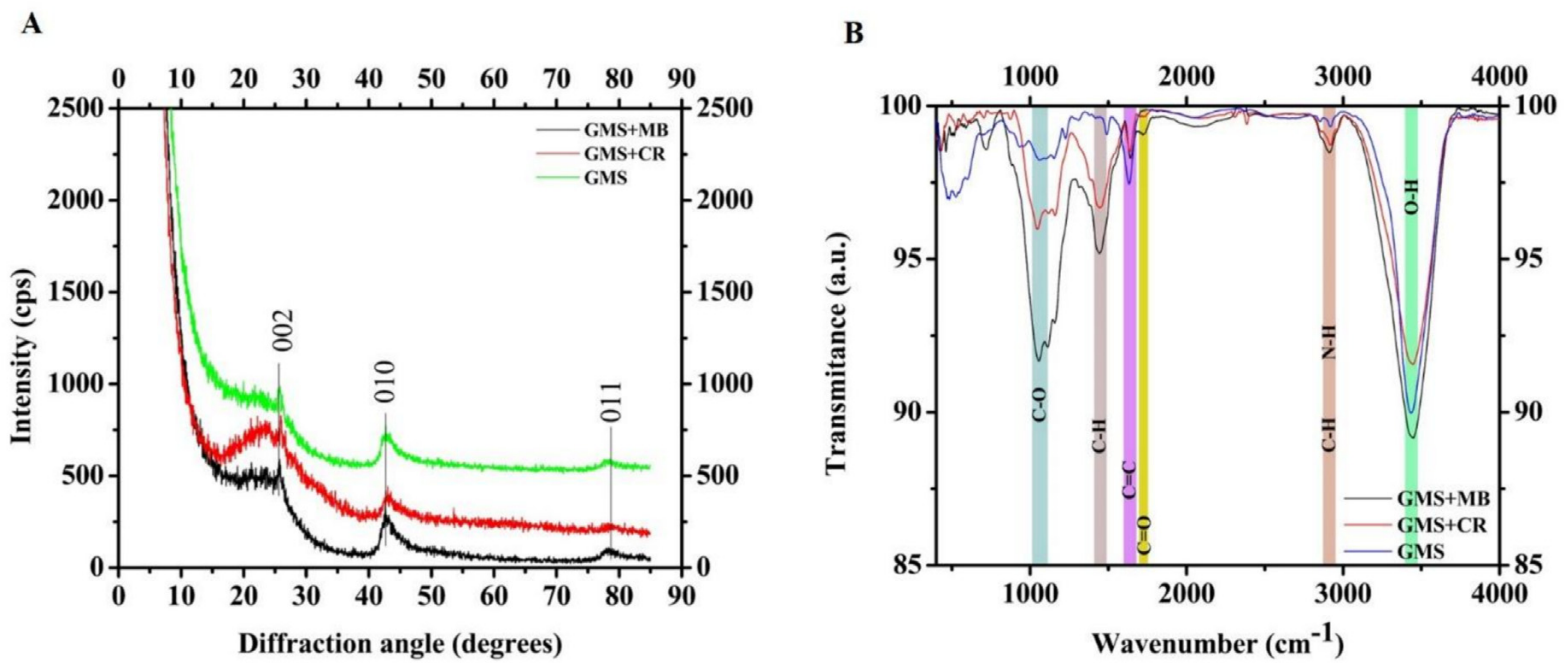
(A) XRD analysis of the adsorbent before and after dye adsorption, (B): FTIR analysis of the GMS before and after the treatment.

Further, XRD analysis was performed to examine the structural changes in GMS due to dye adsorption, which also helps in understanding the type of adsorption. Fig. 8A presents the XRD analysis of GMS before and after treatment with both dyes. X-ray diffraction measurements were conducted using a Philips PW 1830 diffractometer equipped with a Cu anode (operating at 40 kV voltage and 30 mA cathodic current). A Ni filter was used to absorb CuKβ radiation. The diffraction patterns reflect the interaction of X-ray photons with the carbon framework, resulting in (i) a broad and intense X-ray scattering and (ii) three characteristic diffraction bands due to constructive interference within the ordered crystalline structure of GMS. The XRD bands appear at diffraction angles of 26.0°, 42.7°, and 78.5°, corresponding to *d*-spacings of 3.45 Å, 2.11 Å, and 1.22 Å, respectively. Based on previous studies of GMS, these bands correspond to the 002, 10, and 11 crystalline planes [88]. The relative peak intensities do not show significant variation, indicating that dye adsorption does not alter the nano-structural features of the adsorbent. This further supports the occurrence of physisorption, where the dye molecules adhere to the adsorbent surface without affecting its structural integrity.

Additionally, FTIR analysis was performed to confirm the type of adsorption and to understand the arrangement of bonds on the adsorbent surface. Fig. 8B presents the FTIR analysis of GMS before and after dye treatment, following the same methodology. The FTIR spectrum shows the valuable insight into the molecular interactions occurring within the GMS and its interactions with MB and CR. One of the most prominent features is the broad band around 3400 cm^−1^, which corresponds to O–H stretching vibrations. This suggests the presence of hydroxyl groups, possibly from residual water or surface hydroxyl functionalities on the Graphene framework, which are involved in hydrogen bonding (notable changes in the left shoulder of the peak). Another key feature appears around 2950 cm^−1^, which is associated with C–H stretching vibrations, likely due to alkyl groups from any surface functionalization or residual organic species on the graphene structure.

After dye’s adsorption, the peak position is red shifted by approximately 12 cm^−1^, and its intensity decreased, indicating involvement of the C–H and N–H bonds in the interaction. The strong absorption at 1740 cm^−1^ corresponds to C=O stretching, indicating the presence of carbonyl groups, which may arise from surface oxidation or functional groups introduced during synthesis. A significant change is observed around 1640 cm^−1^, where the introduction of MB and CR alters the intensity and position (red shift) of this band. This region corresponds to C=C stretching in aromatic rings or conjugated systems, which is a key feature of both graphene structure and the aromatic frameworks of MB and CR. The increased intensity in this region suggests strong interactions between the dyes and the graphene sponge, likely through π–π stacking or electrostatic interactions. Further down the spectrum, bands around 1450 cm^−1^ correspond to C–H bending, while the absorption near 1100 cm^−1^ is associated with C–O stretching vibrations, likely due to oxygen-containing functional groups on the graphene surface. The intensity of this band decreased after the dye’s adsorption, and the position is blue-shifted, indicating some strengthening of this band. These features indicate the presence of structural modifications or surface functionalization, which were induced by the adsorption behavior of MB and CR. When the three spectra were compared, the pure GMS sample served as a baseline, displaying characteristic bands for the oxygenated functional groups and the graphene framework. However, when MB and CR are introduced, significant changes emerge, particularly in the 1640 cm^−1^ region. These shifts suggest that the dyes interact with the mesoporous graphene surface, supposedly through π–π stacking, hydrogen bonding, dispersion interactions and/or stronger electrostatic attractions. Although the core graphene structure remains intact, these interactions highlight the effective adsorption of MB and CR onto the GMS, influencing the overall spectral profile.

### Hydrogen bond analysis and structural stability of hybrid structures

**Fig. 9:**
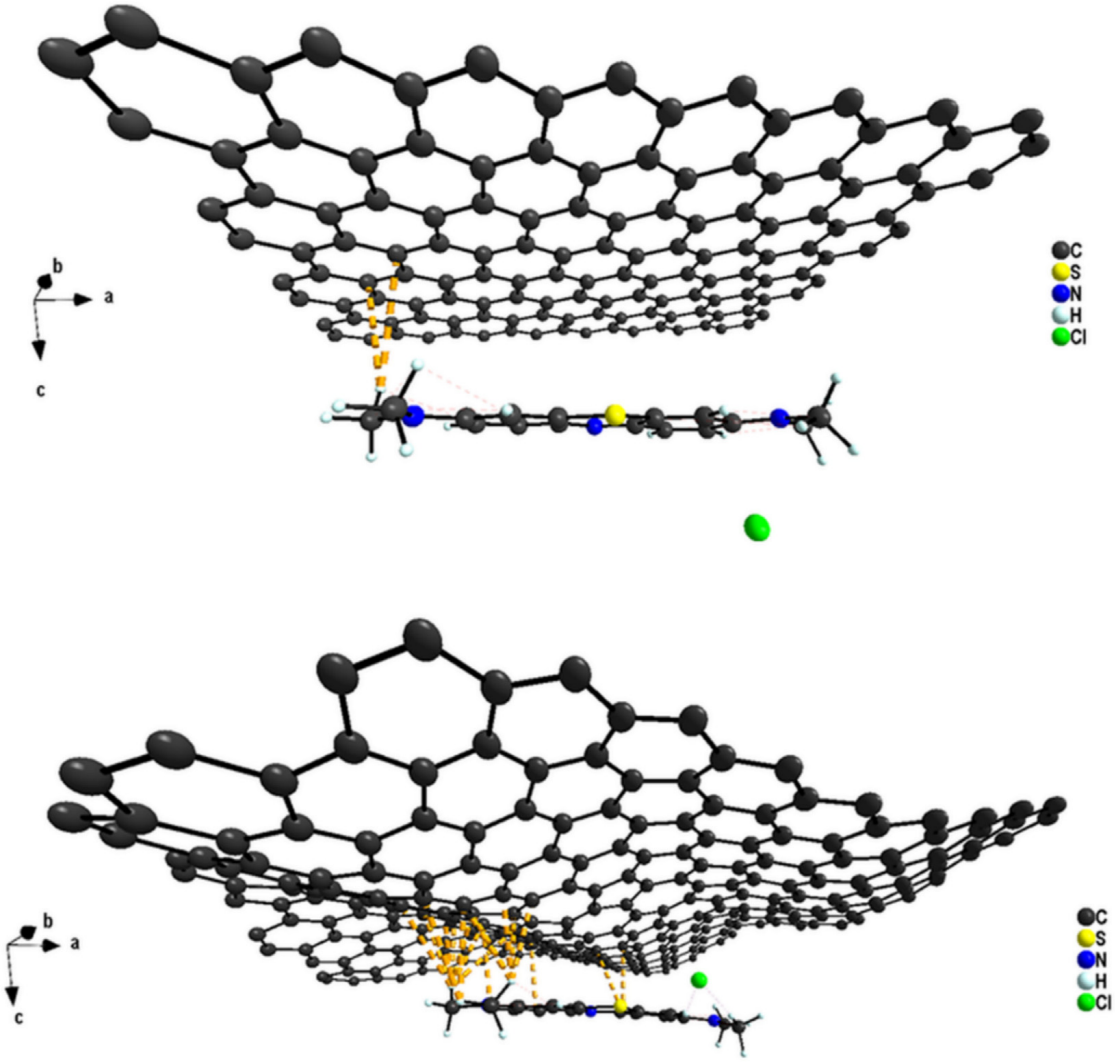
The C–H_MB_⋯C_G6/G57_ hydrogen bonds (gold dash) in the MB-G6 (up) and MB-G57 (down) hybrid structures.

**Fig. 10:**
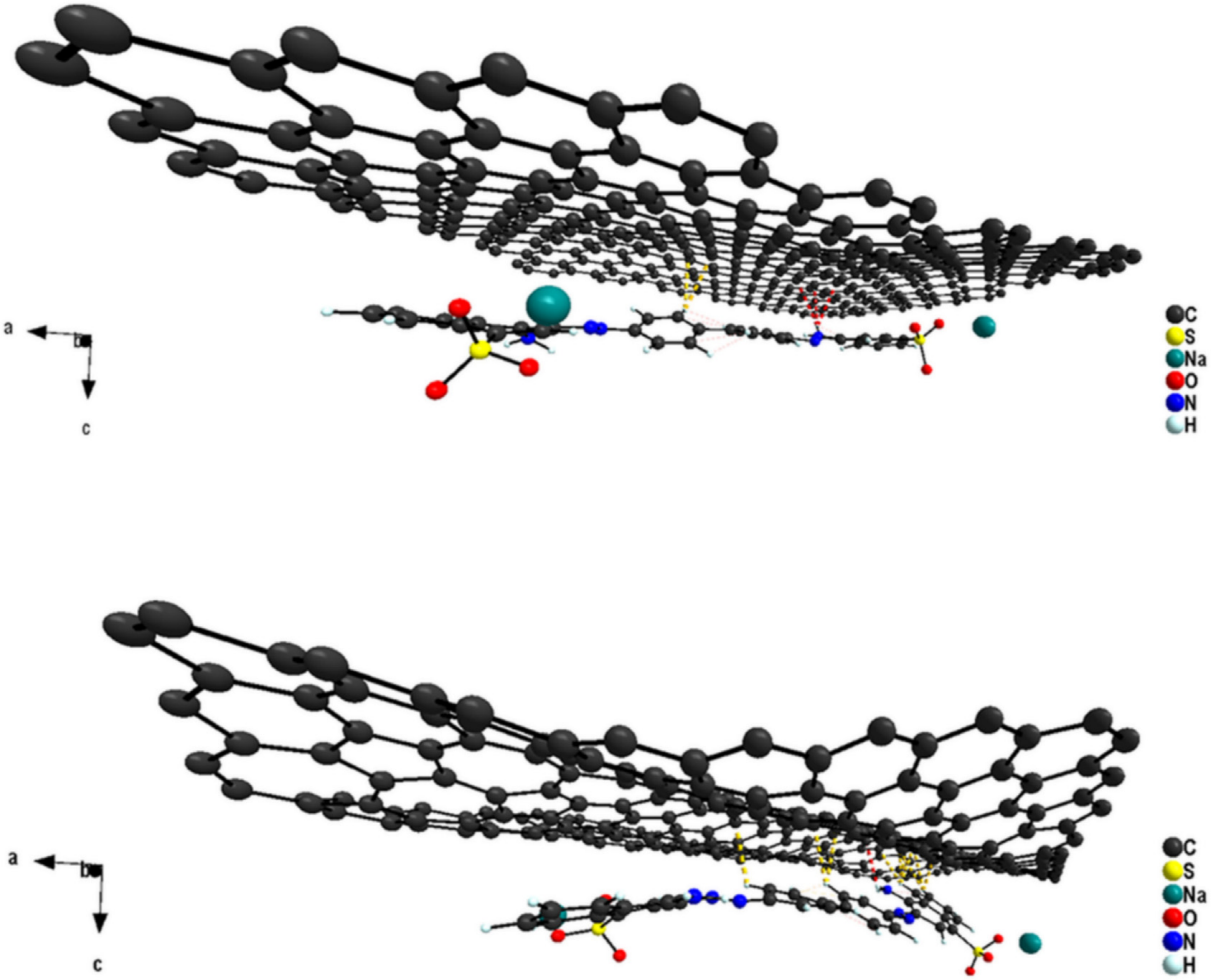
The C-H_CR_⋯C_G6_ (gold dash) and N-H_CR_⋯C_G6/G57_ (red dash) hydrogen bonds in the CR-G6 (up) and CR-G57 (down) hybrid structures.

In the study of the interactions of both azo dyes with the surface of pristine (G6) and modified carbon surface (G57), the focus on hydrogen bonds acting in the structures was conducted. It should be mentioned that, except for hydrogen bonds, also stronger electrostatic, weak van der Waals, π–π and dispersion interactions are present in both structures, supporting the statement mentioned in previous paragraphs. All these mentioned interactions play an important role in the stabilization of the MB/CR-G6/G57 hybrid systems. In the case of G6, with the adsorbed MB dye, the graphene layer remained almost unchanged (Fig. 9, up). On the other hand, the adsorption of CR caused a slightly curved deformation of the graphene sheet (Fig. 10, up). It should be caused by the larger size of the CR molecule and the different distribution of electron density due to a twofold number of heteroatoms and more aromatic rings in the CR molecule than in the MB one, i.e. large number of weak van der Waals, dispersion and π–π interactions are acting in the system. Topological defects in the MB-G57 and CR-G57 model structures caused the waving of the modified graphene surface (Fig. 9, Fig. 10, down) and attraction of azo dye molecules towards these defects, acting as reaction centers due to the redistribution of electron density at the surface compared to the pristine graphene.

The strength of hydrogen bonds was evaluated according to the criteria published by Desiraju & Steiner [76], and the D-H⋯A (D- donor, H- hydrogen, A- acceptor) hydrogen bonds for both azo dyes, as an example, are summarized in Table 4. All these hydrogen bonds are of weak strength. For both systems with adsorbed MB dye, only one type of weak hydrogen bond interaction was identified between the C–H functional group of MB and the carbon atoms of the G6 and/or surface, respectively. In the MB-G6 system, two very weak hydrogen interactions of 3.67 Å and 3.79 Å were identified, which suggests that the adsorption of MB dye on the G6 surface is not so effective, and mainly dispersion and π–π interactions are present in the system. On the other hand, for the system with topological defects, G57, the much more hydrogen bond interactions, of weak strengths, are identified with a median value of 3.26 Å. The MB dye was attracted by topological defects, 5- and 7-membered rings, due to the higher electron density [74]. Adsorption of CR dye on the G6 and G57 surfaces is more effective due to the presence of a larger amount of the weak van der Waals, dispersion and π–π interactions and supported by two different types of hydrogen bonds (Table 5).

**Table 4:** Comparison of the current approach with the available literature.

Dye	Adsorbents	Removal (%)	Time (min)	Concentration (mg/L)	References
MB	Nanocomposite	99	30	10	[25]
MB	MCuOPNs	92.1	20	10	[26]
CR	AgNPs	100	120	5	[34]
CR	Cashew nut shell	98.52	120	20	[84]
MG	ZnONPs-coated cotton fabric	100	120	6.25	[27]
MB	CoFe2O4-yeast	99	120	20	[19]
CR	Mixed Fish Scales	99	60	20	[85]
MB	NC-based aerogels	90	−---	10	[45]
CR	Fly ash (FA)	−---	60	50	[73]
CR	Coagulant (Surjana seed powder)	98	60	25	[86]
CR	Fe_x_ Co_3-x_ O_4_	86.12	−---	10	[67]
CR	ZPG	78.89	480	20	[87]
MB	GMS	100	32	1	This work
CR	GMS	100	35	1	This work

**Table 5:** The D–H⋯A hydrogen bond (minimal; median; maximal) for studied structures.

Models	Bond type	D–H⋯A (Å)
MB-G6	C–H_MB_⋯C_G6_	3.67; 3.79

MB-G57	C–H_MB_⋯C_G57_	2.92; 3.26; 3.68

CR-G6	C–H_CR_⋯C_G6_	2.92; 2.99
	N–H_CR_⋯C_G6_	2.61; 2.85; 2.98

CR-G57	C–H_CR_⋯C_G57_	2.65; 2.82; 2.92
	N–H_CR_⋯C_G57_	2.97

The first type is among the C–H functional groups of CR and the carbon atoms of the G6/G57 surfaces. The second one is among the N–H functional groups and the G6/G57 carbon atoms. All types of interactions are slightly stronger, as in the case of MB-G6/G57 systems, but still of weak strength. For the CR-G6 system, two C–H⋯C hydrogen bond interactions with the value of 2.92 Å and 2.99 Å were identified, as well as three N–H⋯C ones reaching values of 2.61 Å, 2.85 Å and 2.98 Å. Similarly, as in the case of MB-G6, the adsorption of CR dye onto the surface of pristine G6 will not be preferred. Furthermore, modified G57 with adsorbed CR formed more of weak C–H⋯C hydrogen bonds with a median value of 2.82 Å, and only one N–H⋯C hydrogen bond with a value of 2.97 Å. The CR dye was also attracted by more reactive topological defects as in the MB systems.

Intramolecular interactions among the C–H and N–H functional groups and the carbon atoms of both dyes, stabilizing the dye molecules, contributed to the overall stability of all four studied systems. In the figures, these interactions are marked in tiny gold dashes.

The overall structural stability of the studied systems correlates well with the strength of hydrogen bonds and is supported by dispersion and π–π interactions. Calculated adsorption energy (*E*_ads_), according to Equation (1) follows the strength of hydrogen bonds in the systems. For MB dye, the − 41.0 kJ/mol (MB-G6) and − 112.1 kJ/mol (MB-G57), and for CR dye, − 267.7 kJ/mol (CR-G6) and − 292.5 kJ/mol (CR-G57) values clearly favour the G57 structure (GMS material) for effective removing MB and CR azo dyes.

## Reusability of GMS

**Fig. 11:**
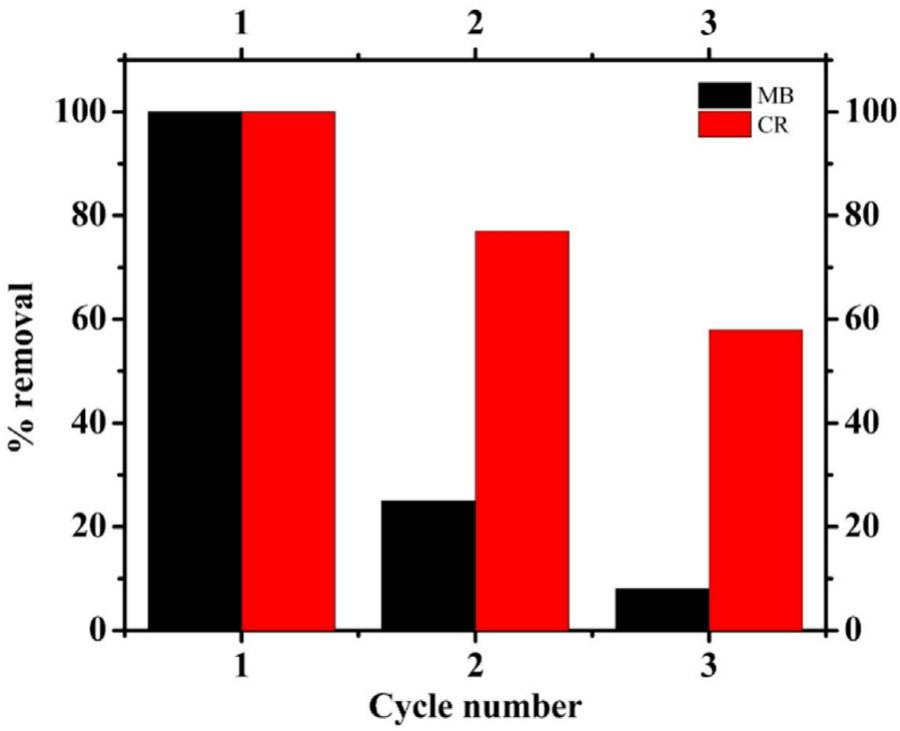
% removal of dyes by recycling of the GMS in the removal process.

The reusability of the GMS catalyst was examined by comparing its ability to reuse with both dyes under identical conditions shown in Fig. 11. After the completion of the first run, the catalyst was recovered through centrifugation and taken directly to the second batch without any additional processing, as shown in the figure below, to evaluate its complete reusability. Two further runs were conducted, and the results showed a consistent loss in the catalytic activity with every reuse. Based on these findings, the GMS catalyst can be effectively used for only one to two (in CR) continuous cycles before performance losses are evident. For a greater number of cycles, catalyst regeneration will be needed to restore its activity level.

## Conclusions

This study demonstrates the effective removal of azo dyes from aqueous solutions using a GMS as an efficient adsorbent. The adsorption of Methylene Blue (MB) and Congo Red (CR) onto GMS was systematically evaluated through experimental and theoretical characterization techniques, confirming the material’s high adsorption capacity and rapid kinetics. The GMS demonstrated the complete adsorption of both azo dyes within a short time frame (30–35 min) with a minimal amount of 1 mg L^−1^, which is in contrast to the findings in other available literature. This advancement provides a step forward for the use of carbon without leaving a large carbon footprint on the environment. The FTIR analysis provided critical insights into the interactions between the dyes and GMS, revealing characteristic peaks at 1640 cm^−1^ associated with C=C functional groups and aromatic π–π interactions, which also play a key role in adsorption, as confirmed also by theoretical modeling of these systems at the analysis of acting interactions in the structures. XRD patterns confirmed the structural integrity of the graphene framework, highlighting the mesoporous nature of GMS, which is unaffected by dye adsorption on the GMS surface. Furthermore, ELS measurements indicated a highly negative zeta potential, suggesting strong electrostatic interactions with cationic MB and effective adsorption of anionic CR through surface functionalization. SEM images further validated the porous architecture of GMS, showing a high surface area with interconnected mesopores that facilitate efficient dye uptake. The adsorption kinetics followed a pseudo-second-order model, indicating chemisorption as the dominant mechanism. Isotherm studies revealed that MB adsorption was best described by the Freundlich model, indicating multilayer adsorption on heterogeneous surface sites, while CR adsorption followed the Temkin model, suggesting adsorption driven by indirect interactions and uniform binding energy distribution across the surface. The different mechanisms were indirectly confirmed also by the values of calculated adsorption energies. The presence of 5, 7-membered rings caused the almost three times stronger adsorption of MB dye and ‘only’ by ∼20 kJ mol^−1^ higher adsorption energy of the CR one in comparison with the pristine graphene. The results obtained demonstrate that GMS is a highly effective and sustainable adsorbent for azo dye removal. The material’s superior adsorption capacity, fast kinetics, and eco-friendly nature make it a promising candidate for wastewater treatment applications. The combination of using both experimental and theoretical approaches for structural characterization and adsorption studies underscores the potential of GMS in mitigating industrial dye pollution, contributing to a cleaner and more sustainable environment.

*Future scope:* The regeneration and reusability of adsorbents play a crucial role in reducing operational costs and enhancing the sustainability of wastewater treatment processes. Preliminary trials have indicated that GMS can be effectively regenerated using ethanol as a solvent following dye adsorption, suggesting its potential for multiple reuse cycles. However, further detailed investigation is required to fully assess the regeneration efficiency, adsorption capacity retention, and long-term stability of GMS under repeated use. Optimizing regeneration conditions, evaluating structural integrity post-regeneration, and exploring alternative eco-friendly desorption methods are recommended as future study priorities. The establishment of a reliable regeneration strategy will not only enhance the economic viability of GMS but also contribute to the minimization of secondary waste generation, thereby further reinforcing its potential as a sustainable adsorbent for large-scale wastewater treatment applications.

## Declaration of Generative AI and AI-assisted technologies in the writing process

Grammar correction and OpenAI tools were used during the preparation of this work to improve the readability and language of the manuscript. The authors review and edit the content as needed and take full responsibility for the published article's content.

## CRediT authorship contribution statement

**Amrita Jain:** Conceptualization, Methodology, Supervision, Validation, Visualization, Writing – original draft, Writing – review & editing, Funding acquisition. **Chandini Kumar:** Formal analysis, Validation, Investigation, Writing – review & editing. **Peter Škorňa:** Formal analysis, Investigation, Visualization, Validation, Writing – original draft, Writing – review & editing. **Hirotaka Nakatsuji:** Investigation, Writing – review & editing. **Hirotomo Nishihara:** Supervision, Writing – original draft, Writing – review & editing, Funding acquisition. **Tamas Szabo:** Investigation, Writing – review & editing, Funding acquisition. **Olena Ivashchenko:** Investigation. **Monika Michalska:** Conceptualization, Funding acquisition, Writing – review & editing. **Eva Scholtzova:** Conceptualization, Methodology, Supervision, Validation, Visualization, Writing – original draft, Writing – review & editing, Funding acquisition.

## Declaration of competing interest

The authors declare that they have no known competing financial interests or personal relationships that could have appeared to influence the work reported in this paper.
